# Multi-omics insights into SERPINE2-driven post-metastatic microenvironmental reprogramming in advanced cancers: implications for molecular targeting and immunotherapy

**DOI:** 10.3389/fimmu.2026.1815463

**Published:** 2026-04-22

**Authors:** Ying Yi, Jianhong Lai, Peng Zhang, Rong Zhang

**Affiliations:** 1 Department of Musculoskeletal Cancer Surgery, Sichuan Clinical Research Center for Cancer, Sichuan Cancer Hospital & Institute, Sichuan Cancer Center, University of Electronic Science and Technology of China, Chengdu, China; 2 Department of Pharmacy, Chengdu integrated TCM&Western medicine hospital, Chengdu, China

**Keywords:** immune evasion, metastatic microenvironment, multi-omics, SerpinE2, therapeutic resistance

## Abstract

Metastasis remains the primary cause of cancer-related mortality, driven not only by intrinsic genetic alterations but also by dynamic reprogramming of the post-metastatic microenvironment. Recent advances in multi-omics technologies—including single-cell transcriptomics, spatial profiling, methylome analysis, chromatin architecture mapping, and proteomics—have reshaped our understanding of metastatic ecosystems and revealed key regulatory nodes that coordinate tumor progression. Among these, SERPINE2 has emerged as a convergent mediator across diverse cancer types. Multi-layered evidence demonstrates that SERPINE2 integrates tumor-intrinsic signaling and microenvironmental remodeling. Mechanistically, SERPINE2 stabilizes oncogenic receptors such as EGFR by inhibiting ubiquitination-dependent degradation, sustains downstream STAT3/ERK activation, and enhances DNA damage response through ATM phosphorylation and homologous recombination repair. Concurrently, as a secreted factor, SERPINE2 promotes cancer-associated fibroblast activation, M2 macrophage polarization, extracellular matrix remodeling, and angiogenesis, collectively fostering immune suppression and metastatic persistence. These dual tumor-intrinsic and stromal functions position SERPINE2 as a molecular hub in post-metastatic adaptation. Targeting SERPINE2 may therefore represent a paradigm shift in advanced cancer therapy by simultaneously disrupting oncogenic persistence, genome maintenance, and immune exclusion. This review synthesizes multi-omics insights into SERPINE2-driven microenvironmental reprogramming and discusses emerging therapeutic strategies integrating molecular targeting and immunotherapy in metastatic cancers.

## Highlights

- Multi-omics analyses consistently identify SERPINE2 as a regulator of metastatic progression and microenvironmental remodeling.
- SERPINE2 coordinates receptor stabilization, DNA damage response, stromal activation, and immune suppression in advanced cancers.
- Targeting SERPINE2 may enhance molecular therapies and immunotherapy by reversing oncogenic persistence and immune exclusion.

## 1 Introduction

Metastasis remains the leading cause of cancer-related mortality worldwide, accounting for the vast majority of deaths in patients with advanced solid tumors (1). Despite substantial progress in targeted therapy and immunotherapy, durable responses are frequently undermined by the complex biological adaptations that occur after tumor dissemination (2, 3). Increasing evidence suggests that metastatic progression is not merely a consequence of intrinsic genetic alterations within cancer cells, but rather a dynamic process driven by bidirectional interactions between disseminated tumor cells and the post-metastatic microenvironment (4, 5). This reprogrammed microenvironment—characterized by stromal remodeling, immune suppression, angiogenesis, and metabolic adaptation—plays a pivotal role in therapeutic resistance and immune evasion.

In recent years, multi-omics technologies have fundamentally reshaped our understanding of metastatic ecosystems (6). Single-cell RNA sequencing (scRNA-seq) has uncovered heterogeneous malignant cell states associated with invasion and organ colonization (7, 8). Spatial transcriptomics has revealed how tumor and stromal compartments are spatially organized to support immune exclusion. Epigenomic profiling, including DNA methylation and chromatin conformation analyses, has illuminated regulatory mechanisms that sustain metastatic gene programs (9, 10). Proteomic and secretome analyses have further identified extracellular mediators that coordinate signaling persistence and stromal communication. Together, these integrative approaches have shifted the paradigm from single-gene models to network-level regulators that orchestrate metastatic adaptation. Among these emerging regulators, SERPINE2 (also known as protease nexin-1) has gained increasing attention across multiple cancer types (11–13). Originally characterized as a secreted serine protease inhibitor, SERPINE2 is now recognized as a multifunctional mediator of tumor progression. Multi-omics evidence has consistently identified SERPINE2 as upregulated in advanced and metastatic tumors, where it functions at the intersection of tumor-intrinsic signaling and microenvironmental remodeling. Mechanistically, SERPINE2 promotes receptor stabilization by inhibiting c-Cbl–mediated ubiquitination of EGFR, thereby sustaining oncogenic signaling cascades such as STAT3 and ERK (11). It enhances DNA damage response signaling through activation of ATM, contributing to radiotherapy resistance and survival under genotoxic stress. In parallel, SERPINE2 facilitates epithelial–mesenchymal transition (EMT), invasion, and dissemination. Importantly, as a secreted factor, SERPINE2 actively reshapes the tumor microenvironment by promoting cancer-associated fibroblast (CAF) activation, macrophage polarization toward an M2 immunosuppressive phenotype, and angiogenic remodeling.

These convergent functions position SERPINE2 as more than a downstream effector; rather, it may serve as a metastatic hub integrating oncogenic signaling persistence with stromal and immune reprogramming in advanced cancers. In this review, we move beyond descriptive summaries of SERPINE2 expression and function and instead position SERPINE2 as a cross-platform regulatory node in post-metastatic ecosystem remodeling. Distinct from prior literature that has largely discussed SERPINE2 in isolated tumor contexts or single mechanistic pathways, this review integrates transcriptomic, single-cell, spatial, epigenetic, chromatin, and proteomic evidence to define how SERPINE2 links malignant cell plasticity, stromal activation, immune exclusion, and therapy adaptation across advanced cancers. We further propose a multi-omics-informed therapeutic framework in which SERPINE2 serves not only as a biomarker of metastatic aggressiveness but also as a candidate target for combination strategies involving molecular therapy and immunotherapy.

## 2 Multi-omics identification ofSERPINE2 in metastatic progression

A defining feature of SERPINE2 in advanced cancers is its recurrent identification across independent omics platforms and tumor types. Rather than emerging from a single discovery pipeline, SERPINE2 has been consistently rediscovered through transcriptomic, epigenomic, chromatin-level, and proteomic analyses of metastatic disease. This cross-platform convergence strongly supports its functional relevance in metastatic progression and post-dissemination adaptation.

### 2.1 Transcriptomic and single-cell evidence

Bulk transcriptomic profiling initially revealed elevated SERPINE2 expression in multiple advanced solid tumors. However, the advent of single-cell RNA sequencing (scRNA-seq) has provided higher-resolution insights into its cellular origin and functional context. In advanced renal cell carcinoma (RCC), integrated scRNA-seq analyses identified SERPINE2 enrichment within malignant cell clusters associated with metastatic potential (12). These SERPINE2-high tumor subpopulations exhibited transcriptional programs linked to epithelial–mesenchymal transition (EMT), hypoxia adaptation, extracellular matrix remodeling, and invasive phenotypes. Importantly, trajectory and state-transition analyses suggested that SERPINE2 expression aligns with dynamic malignant cell states rather than static differentiation markers, indicating a role in metastatic plasticity. Single-cell analyses have also clarified the stromal contribution of SERPINE2. In gastric cancer, large-scale integration of bulk and single-cell transcriptomic datasets demonstrated that SERPINE2 is highly expressed in specific cancer-associated fibroblast (CAF) subtypes enriched in immunosuppressive tumors (14). These CAF populations displayed signatures associated with extracellular matrix deposition, T-cell exclusion, and immune checkpoint resistance. The identification of SERPINE2 as a CAF-derived secreted mediator underscores its dual role as both a tumor-intrinsic factor and a microenvironmental regulator.

Across datasets, SERPINE2 expression consistently correlates with EMT-related transcription factors, matrix remodeling genes, and invasion-associated gene programs. Such transcriptomic convergence suggests that SERPINE2 participates in metastasis not merely as a downstream marker, but as an active component of invasive and organ-colonizing programs.

### 2.2 Epigenetic and methylation regulation

Beyond transcriptional upregulation, multi-omics analyses have uncovered epigenetic mechanisms governing SERPINE2 expression. In hepatocellular carcinoma (HCC), integrative analyses combining RNA sequencing with DNA methylation profiling revealed hypomethylation of the SERPINE2 promoter region in metastatic tumors (11). Functional experiments demonstrated that DNMT1-mediated methylation suppresses SERPINE2 transcription, whereas promoter hypomethylation results in its depression and upregulation. These findings highlight SERPINE2 as an epigenetically regulated gene activated during tumor progression. The epigenetic deregulation of SERPINE2 may reflect broader chromatin remodeling events that accompany metastatic transformation. Indeed, chromatin architecture analyses in metastatic models have suggested that genomic regions harboring metastasis-associated genes—including SERPINE2—can undergo compartment switching toward transcriptionally active chromatin states. Although further validation is required, such structural reorganization provides a plausible mechanistic basis for sustained SERPINE2 activation in advanced disease. Together, methylome and chromatin-level data position SERPINE2 within an epigenetically permissive landscape characteristic of metastatic progression, linking DNA methylation loss and chromatin remodeling to pro-metastatic gene expression.

### 2.3 Proteomic and secretome profiling

Proteomic and secretome analyses further reinforce the functional importance of SERPINE2 in metastatic ecosystems. As a secreted serine protease inhibitor (serpin), SERPINE2 is readily detectable in the extracellular milieu, distinguishing it from many intracellular metastasis regulators. Proteomic studies have identified elevated SERPINE2 protein levels in tumor tissues and circulation in several cancers. In lung cancer, increased serum SERPINE2 levels have been associated with disease progression and therapeutic response, suggesting its potential utility as a minimally invasive biomarker (13). Secretome profiling indicates that SERPINE2 participates in extracellular matrix remodeling, stromal signaling, and intercellular communication within the tumor microenvironment. Importantly, functional proteomic analyses have linked SERPINE2 to signaling networks governing receptor stability and DNA damage response. In hepatocellular carcinoma, SERPINE2 interferes with c-Cbl-mediated ubiquitination of EGFR, thereby stabilizing receptor signaling (11). In lung cancer, SERPINE2 enhances ATM phosphorylation and homologous recombination repair machinery (13). These mechanistic insights bridge proteomic observations with intracellular signaling persistence and therapeutic resistance.

Collectively, transcriptomic, single-cell, epigenetic, chromatin, and proteomic datasets converge on a consistent conclusion: SERPINE2 is recurrently upregulated and functionally implicated in metastatic progression across diverse tumor types. Its identification across multiple omics layers strengthens the argument that SERPINE2 is not a context-specific bystander, but rather a cross-platform metastatic regulator. This multi-omics convergence provides a robust foundation for conceptualizing SERPINE2 as a central mediator of post-metastatic microenvironmental reprogramming and a promising candidate for molecular targeting in advanced cancers.

### 2.4 Critical synthesis of multi-omics evidence

While the repeated identification of SERPINE2 across independent omics platforms strongly supports its relevance in metastatic progression, the evidentiary contributions of these modalities are not equivalent and should be interpreted in a complementary rather than interchangeable manner. Bulk transcriptomic analyses primarily establish recurrent association, showing that SERPINE2 is consistently enriched in advanced, metastatic, or therapy-resistant tumors. However, such datasets are inherently limited in cellular resolution and cannot distinguish whether SERPINE2 expression arises predominantly from malignant cells, fibroblasts, or other stromal components (12). Single-cell transcriptomic approaches provide a critical advance by resolving the cellular origin and state-specific context of SERPINE2 expression. In this regard, scRNA-seq data are particularly valuable for distinguishing tumor-intrinsic SERPINE2-high malignant programs from stromal SERPINE2 enrichment in specific CAF populations, thereby refining biological interpretation beyond bulk-level association. Nevertheless, single-cell data remain largely inferential with respect to function, and the identification of SERPINE2-high cell states does not by itself establish causal roles in metastatic colonization or immune remodeling (12, 14).

Epigenetic and chromatin-level analyses contribute a different layer of evidence by suggesting that SERPINE2 upregulation is not merely a passive transcriptional correlate, but may be supported by stable regulatory alterations such as promoter hypomethylation or permissive chromatin organization. These datasets therefore strengthen the argument that SERPINE2 activation is biologically programmed during tumor progression. Even so, they do not necessarily demonstrate that SERPINE2 is a dominant driver of metastatic behavior, because regulatory activation may also reflect broader state transitions occurring in aggressive tumors. Proteomic and secretome-based evidence is especially important in the case of SERPINE2 because it directly supports its identity as an extracellular effector molecule rather than only a transcript-level marker. This layer of evidence is particularly relevant for understanding how SERPINE2 may mediate stromal communication, extracellular matrix remodeling, macrophage polarization, and immune exclusion. However, protein detection alone still does not establish mechanistic indispensability, and must be integrated with functional perturbation studies.

Taken together, the major strength of the multi-omics literature lies in cross-platform convergence: transcriptomics supports recurrent association, single-cell analyses clarify cellular source and malignant or stromal states, epigenetic and chromatin studies provide regulatory plausibility, and proteomics supports extracellular functional relevance. Collectively, these modalities do not offer the same type of proof, but they converge to position SERPINE2 as a biologically credible and context-dependent regulator of metastatic progression. At the same time, the current body of evidence remains strongest for convergence and plausibility, whereas definitive causality still depends on targeted experimental validation and clinical corroboration.

## 3 SERPINE2 as a driver of post-metastatic microenvironmental reprogramming

Metastatic colonization requires cancer cells to survive detachment-induced stress, adapt to foreign tissue environments, evade immune surveillance, and withstand therapeutic pressure (15, 16). Increasing evidence suggests that SERPINE2 contributes to this complex adaptation process at multiple levels. Rather than acting solely as a secreted protease inhibitor, SERPINE2 operates as a multifunctional regulator that integrates oncogenic signaling persistence, genome maintenance, and stromal reprogramming. These coordinated activities position SERPINE2 as a central driver of post-metastatic microenvironmental remodeling.

### 3.1 Stabilization of oncogenic signaling in metastatic cells

A critical requirement for metastatic survival is the sustained activation of growth and survival signaling pathways after dissemination (17, 18). One of the most mechanistically defined roles of SERPINE2 lies in its ability to promote receptor stabilization through interference with ubiquitin-mediated degradation. In hepatocellular carcinoma, SERPINE2 directly interacts with the intracellular domain of epidermal growth factor receptor (EGFR) and disrupts its association with the E3 ubiquitin ligase c-Cbl. Under physiological conditions, c-Cbl recognizes phosphorylated EGFR and mediates its ubiquitination, triggering receptor internalization and lysosomal degradation. SERPINE2 interferes with this process, thereby reducing EGFR ubiquitination and preventing receptor turnover (11). This mechanism represents a form of “ubiquitination escape,” allowing EGFR to evade proteostatic regulation. As a consequence, EGFR protein levels are stabilized, leading to sustained activation of downstream oncogenic cascades, including STAT3 and ERK signaling. Persistent activation of these pathways enhances proliferative capacity, promotes invasion, and supports survival under stress conditions encountered in distant organs. In the post-metastatic niche—where nutrient availability, oxygen tension, and immune pressure differ from the primary tumor—such oncogenic persistence may be critical for successful colonization. Importantly, receptor stabilization by SERPINE2 highlights a broader concept: metastatic cells may rely not only on genetic mutations but also on post-translational regulatory mechanisms to maintain signaling intensity. By modulating ubiquitination-dependent receptor degradation, SERPINE2 provides metastatic cells with a non-mutational strategy to amplify growth signals.

### 3.2 Activation of DNA damage response and therapy adaptation

In addition to sustaining proliferative signaling, metastatic cells must preserve genome integrity while adapting to, replication stress, and therapeutic challenge (19–22). Emerging evidence, primarily from lung cancer models, suggests that SERPINE2 may participate in the DNA damage response (DDR), particularly in the context of radiotherapy adaptation. Mechanistically, SERPINE2 has been reported to promote ATM phosphorylation and support the accumulation of homologous recombination-associated repair factors, including RAD51, thereby facilitating the repair of DNA double-strand breaks and limiting genotoxic damage. In these models, SERPINE2 depletion impaired homologous recombination efficiency, reduced radioresistance, and was accompanied by attenuated DDR signaling, supporting a functional link between SERPINE2 and genome maintenance under therapeutic stress (13).

Importantly, however, direct mechanistic validation of the SERPINE2–ATM–RAD51 axis remains limited and is currently concentrated in lung cancer systems. Therefore, this pathway should be interpreted as an experimentally supported but not yet universally established mechanism across metastatic cancers. Even so, the available evidence raises the possibility that SERPINE2 contributes to therapy adaptation not only by sustaining survival signaling but also by reinforcing DNA repair competence under adverse conditions. This interpretation is further supported by the broader association of SERPINE2 with aggressive disease behavior, metastatic progression, and therapy-resistant phenotypes across multiple tumor contexts (23).

A particularly intriguing possibility is that SERPINE2 may function as a molecular bridge between receptor-driven oncogenic programs and genome-protective stress responses. Because EGFR signaling has been implicated in cell survival, replication-associated stress adaptation, and treatment resistance, persistent receptor stabilization mediated by SERPINE2 may indirectly create a permissive context for stronger DDR activation. In this sense, SERPINE2 may coordinate two complementary survival axes in metastatic cells: sustained growth signaling and enhanced genomic stability. Rather than representing an isolated repair factor, SERPINE2 may therefore act as a broader adaptive integrator that helps metastatic cells endure colonization-associated stress and therapeutic exposure. At the same time, future studies combining clinical cohorts with single-cell and spatially resolved multi-omics will be important to determine in which tumor cell states and stromal niches this DDR-supportive role is most relevant (12).

### 3.3 Remodeling the metastatic microenvironment

**Figure 1:**
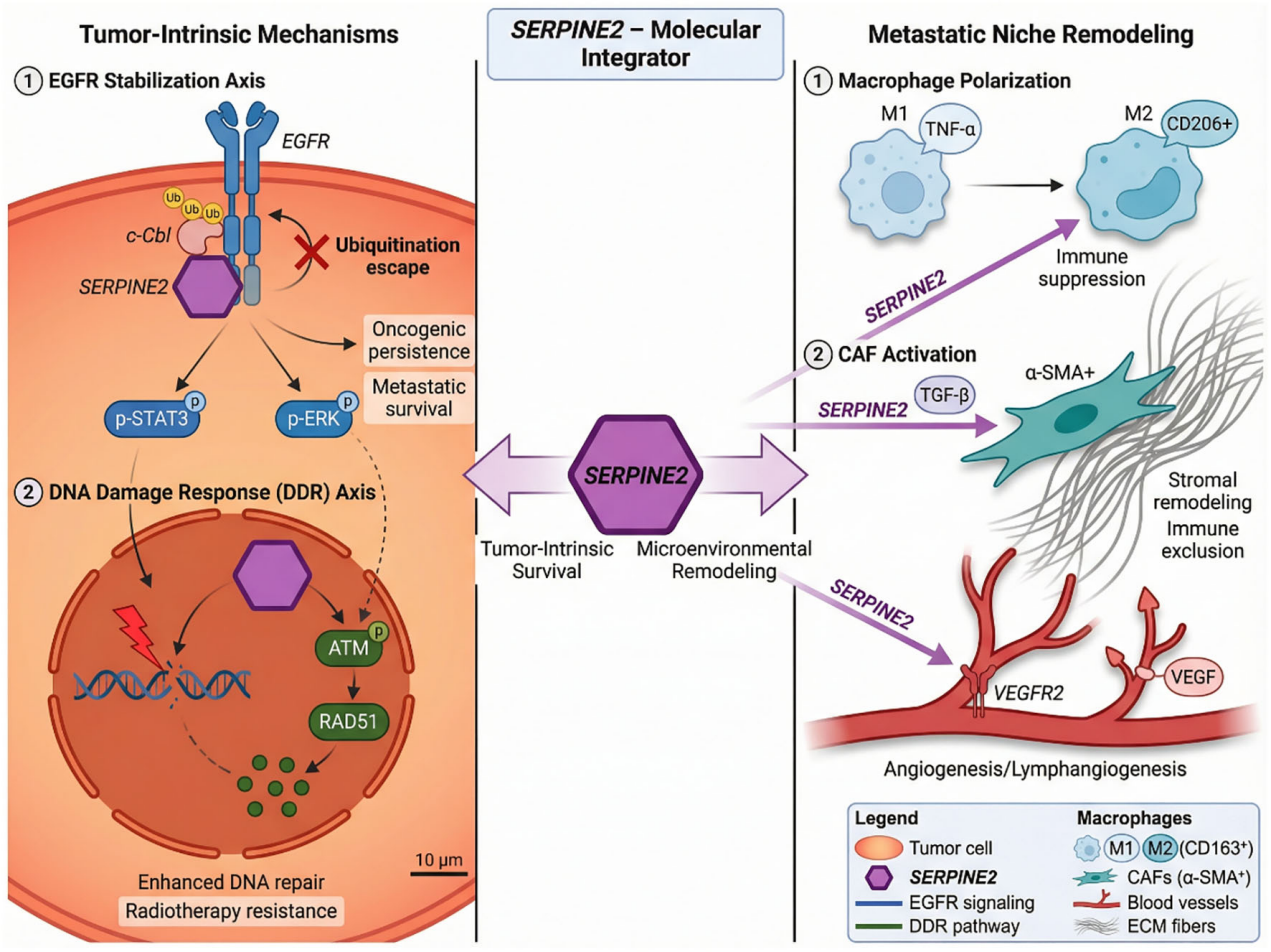
SERPINE2-driven post-metastatic microenvironmental reprogramming. SERPINE2 stabilizes EGFR by inhibiting c-Cbl–mediated ubiquitination, sustaining STAT3/ERK signaling and oncogenic persistence. Concurrently, SERPINE2 enhances ATM activation and RAD51 recruitment, promoting DNA repair and therapy resistance. As a secreted factor, SERPINE2 induces M2 macrophage polarization, activates cancer-associated fibroblasts, and stimulates angiogenesis, collectively reshaping the metastatic niche and facilitating immune evasion and tumor colonization.

Beyond tumor-intrinsic functions, SERPINE2 actively participates in reshaping the metastatic microenvironment. As a secreted protein, SERPINE2 exerts paracrine effects on stromal and immune compartments, contributing to immune suppression and niche remodeling. In colorectal cancer, tumor-derived SERPINE2 promotes macrophage polarization toward an M2 phenotype. M2-like tumor-associated macrophages (TAMs) are characterized by immunosuppressive and pro-tumorigenic functions, including secretion of anti-inflammatory cytokines, extracellular matrix remodeling enzymes, and growth factors (24). SERPINE2-induced M2 polarization establishes a positive feedback loop in which macrophages further enhance tumor proliferation, invasion, and survival. This macrophage reprogramming contributes to immune evasion within the metastatic niche. In gastric cancer, multi-omics analyses have identified SERPINE2 as highly enriched in specific cancer-associated fibroblast (CAF) subtypes associated with poor prognosis and immunotherapy resistance. CAF-derived SERPINE2 participates in extracellular matrix remodeling and stromal activation, potentially contributing to immune exclusion and impaired T-cell infiltration. These findings highlight SERPINE2 as a mediator of stromal reorganization in advanced tumors. Additionally, in oral squamous cell carcinoma, SERPINE2 has been implicated in promoting angiogenesis and lymphangiogenesis. By facilitating vascular and lymphatic network expansion, SERPINE2 supports nutrient supply, metastatic dissemination, and tumor cell trafficking. Enhanced vascular remodeling further reinforces the establishment of a supportive metastatic microenvironment. Taken together, these observations support a unifying concept: SERPINE2 functions both as a tumor-intrinsic regulator and as a stromal reprogramming factor. It strengthens oncogenic persistence within cancer cells while simultaneously orchestrating immune suppression, fibroblast activation, and vascular remodeling in the surrounding niche. This dual role underscores its central position in post-metastatic microenvironmental reprogramming and provides a compelling rationale for targeting SERPINE2 in advanced cancers. **Figure 1** illustrates how SERPINE2 integrates tumor-intrinsic survival signaling with stromal and immune reprogramming in the post-metastatic niche. By coordinating receptor stabilization, genome maintenance, and microenvironmental remodeling, SERPINE2 promotes metastatic persistence and therapeutic resistance.

## 4 SERPINE2 and immune evasion in advanced cancers

Immune evasion is a defining feature of advanced and metastatic cancers and represents a major obstacle to durable therapeutic responses (25, 26). Increasing evidence suggests that SERPINE2 contributes to immune escape not only through tumor-intrinsic signaling pathways but also by reshaping the cellular and structural components of the tumor microenvironment. By influencing macrophage polarization, fibroblast activation, extracellular matrix organization, and oncogenic signaling persistence, SERPINE2 may help establish an immunologically permissive niche that undermines effective antitumor immunity. At present, however, the strongest SERPINE2-specific immune evidence is concentrated in myeloid and stromal compartments, particularly macrophage polarization, CAF-associated remodeling, and extracellular matrix reorganization. By contrast, direct evidence linking SERPINE2 to T-cell exhaustion phenotypes or immune checkpoint resistance remains relatively limited and should be interpreted more cautiously.

One of the most compelling lines of evidence linking SERPINE2 to immune suppression comes from its role in macrophage reprogramming. In colorectal cancer models, tumor-derived SERPINE2 promotes the polarization of macrophages toward an M2-like phenotype characterized by increased expression of immunosuppressive mediators such as IL-10 and ARG1 (24). M2-polarized tumor-associated macrophages (TAMs) suppress cytotoxic T-cell responses, secrete pro-tumorigenic growth factors, and facilitate extracellular matrix remodeling. Through this polarization shift, SERPINE2 indirectly dampens adaptive immune responses while reinforcing a protumoral inflammatory environment. The resulting macrophage-rich, immunosuppressive niche can support metastatic colonization and limit immune-mediated tumor clearance. Beyond macrophage regulation, SERPINE2 also appears to influence immune evasion through its association with cancer-associated fibroblasts (CAFs). Multi-omics analyses in gastric cancer have identified SERPINE2 as highly enriched in specific CAF subpopulations linked to poor prognosis and immunotherapy resistance. These CAF subsets exhibit transcriptional programs associated with extracellular matrix deposition, stromal stiffening, and reduced immune cell infiltration. In this context, SERPINE2 may function as a mediator of stromal remodeling that indirectly limits T-cell penetration into tumor parenchyma. Importantly, this interpretation is currently supported more by stromal and matrix-associated immune exclusion patterns than by direct SERPINE2-focused demonstrations of T-cell dysfunction or exhaustion. Thus, the available data are stronger for impaired immune access than for a fully established SERPINE2-specific T-cell exhaustion program. The accumulation of dense extracellular matrix components can create both physical and biochemical barriers that impede immune cell trafficking and promote immune exclusion, a phenotype frequently observed in non-responsive tumors treated with immune checkpoint inhibitors. High SERPINE2 expression is often associated with transcriptional signatures indicative of reduced cytotoxic T-cell infiltration and enrichment of stromal activation pathways. Although direct mechanistic studies are still needed in certain tumor types, the convergence of macrophage polarization, fibroblast activation, and matrix remodeling strongly suggests that SERPINE2 contributes to the establishment of an immune “cold” microenvironment. Moreover, sustained activation of EGFR–STAT3 signaling driven by SERPINE2-mediated receptor stabilization may further exacerbate immune suppression at the tumor-intrinsic level. STAT3 is known to regulate immunosuppressive cytokine production and impair antigen presentation, thereby linking oncogenic signaling persistence to immune evasion (27–30). These observations raise the possibility that SERPINE2 may influence responsiveness to immune checkpoint blockade. Tumors characterized by dense stromal barriers, dominant M2 macrophage infiltration, and limited CD8^+^ T-cell access frequently exhibit primary or acquired resistance to anti-PD-1/PD-L1 therapies. In such settings, SERPINE2-driven microenvironmental reprogramming could represent an upstream determinant of checkpoint therapy failure. Although prospective clinical validation remains necessary, the multi-omics convergence of stromal activation and immune exclusion signatures in SERPINE2-high tumors provides a strong rationale for further investigation.

**Figure 2:**
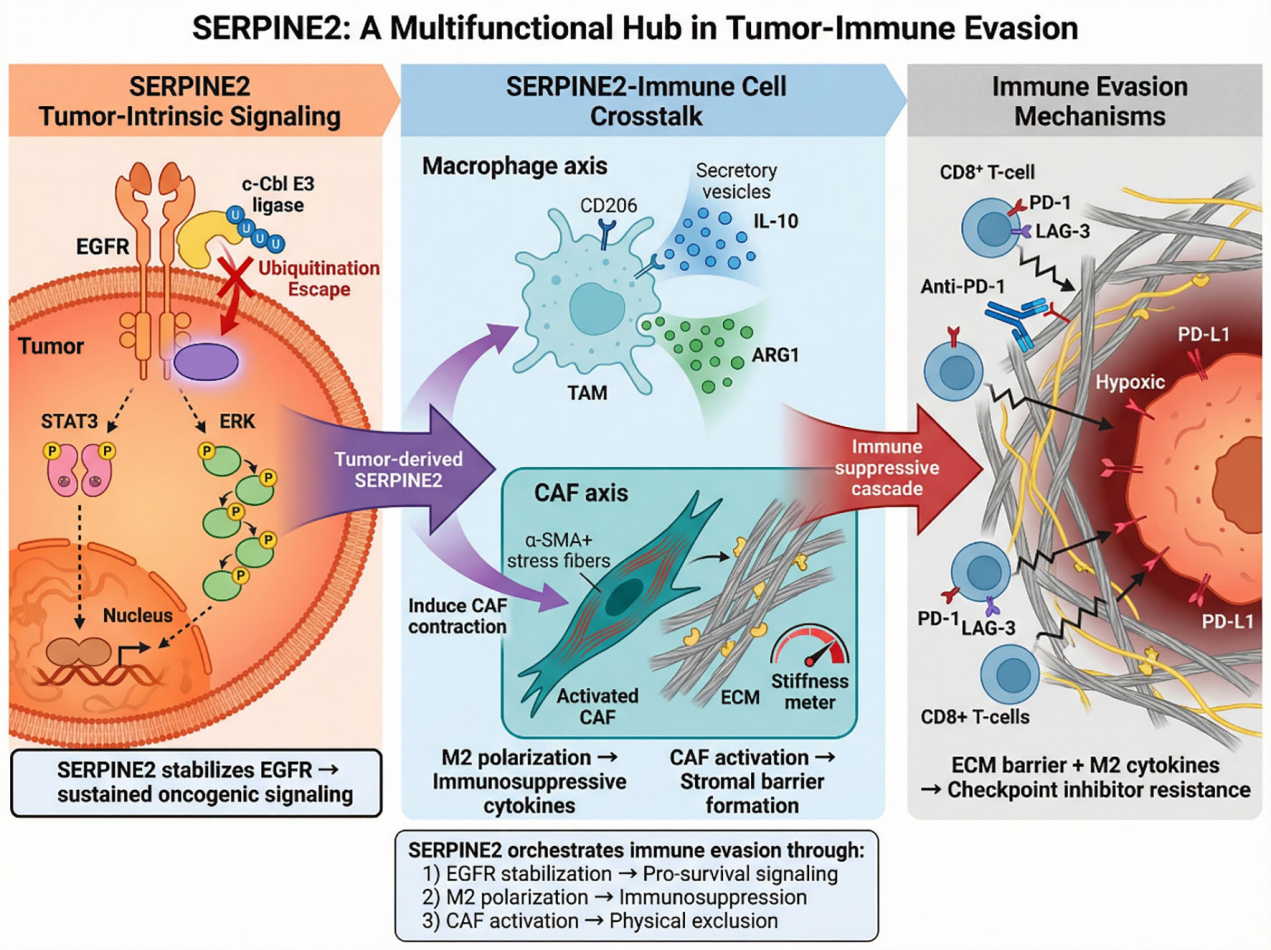
SERPINE2-mediated immune evasion in advanced cancers. SERPINE2 stabilizes EGFR in tumor cells, promoting sustained oncogenic signaling. It drives immune evasion by inducing M2 macrophage polarization, activating CAFs, and remodeling the extracellular matrix (ECM). This reprogramming creates a physical and biochemical barrier that limits T-cell infiltration, contributing to immune exclusion and resistance to immune checkpoint inhibitors.

Overall, the current evidence supports a SERPINE2-centered immune-evasion framework most strongly at the level of myeloid reprogramming, stromal activation, and matrix-mediated immune exclusion. By comparison, direct SERPINE2-specific evidence for T-cell exhaustion, checkpoint resistance, or causal immunotherapy non-response remains less developed. These latter aspects should therefore be considered emerging hypotheses that warrant dedicated validation in immune profiling, spatial, and clinical response datasets. Taken together, a conceptual model emerges in which SERPINE2 orchestrates immune evasion through extracellular matrix remodeling and stromal-immune reprogramming. By enhancing matrix deposition, activating fibroblasts, and skewing macrophages toward immunosuppressive phenotypes, SERPINE2 may create a barrier that restricts effector T-cell infiltration while simultaneously reinforcing tumor-intrinsic survival signaling. In this framework, SERPINE2 acts as both a signaling stabilizer within cancer cells and a structural architect of the metastatic niche. Targeting SERPINE2 could therefore represent a strategy not only to suppress tumor growth but also to restore immune accessibility and improve the efficacy of immunotherapeutic interventions in advanced cancers. **Figure 2** illustrates how SERPINE2 facilitates immune evasion by stabilizing EGFR, reprogramming macrophages to an M2 phenotype, and promoting stromal remodeling in the metastatic niche. These effects lead to immune exclusion, limiting the effectiveness of immunotherapies and supporting metastatic progression.

Nevertheless, the current evidence should also be interpreted with caution. The biological functions of SERPINE2 are likely to be context-dependent rather than fully uniform across tumor types. In some cancers, SERPINE2 appears to function predominantly as a tumor-intrinsic regulator associated with malignant cell plasticity, receptor signaling persistence, and therapy adaptation, whereas in others its strongest associations are observed in stromal compartments, particularly CAF-rich or macrophage-enriched immunosuppressive niches. In addition, several unresolved questions remain, including the relative contribution of tumor-derived versus stromal-derived SERPINE2, the heterogeneity and possible origins of SERPINE2-enriched CAF populations, and the extent to which tissue-specific regulatory networks shape its downstream effects. These considerations suggest that SERPINE2 should not yet be viewed as a universally conserved effector, but rather as a context-dependent mediator whose dominant functions may vary across tumor ecosystems.

## 5 Therapeutic implications: targeting SERPINE2 in the multi-omics era

### 5.1 Biomarker potential

SERPINE2 exhibits several characteristics that support its development as a clinically relevant biomarker. Elevated tumor expression has been consistently associated with advanced disease stage, metastatic dissemination, and unfavorable survival outcomes across multiple cancer types. Multi-omics profiling further links high SERPINE2 levels with epithelial–mesenchymal transition programs, stromal activation signatures, and immune suppression phenotypes, suggesting that SERPINE2 expression reflects an aggressive, microenvironment-driven tumor state rather than a context-specific alteration. Importantly, as a secreted protein, SERPINE2 is detectable in circulation. Increased serum levels reported in lung cancer patients highlight the potential for non-invasive monitoring. Circulating SERPINE2 could be explored as a dynamic biomarker for metastatic progression, therapeutic response, or minimal residual disease. In addition, functional studies demonstrating that SERPINE2 enhances ATM phosphorylation and homologous recombination repair suggest that its expression level may predict radiotherapy responsiveness. Tumors with high SERPINE2 activity may display increased DNA repair capacity and relative resistance to genotoxic therapies. Similarly, in cancers where SERPINE2 stabilizes EGFR and sustains downstream signaling, elevated expression may correlate with reduced sensitivity to tyrosine kinase inhibitors. Thus, integrating SERPINE2 status into multi-omics-based stratification frameworks may improve patient selection for targeted and radiation-based treatments.

### 5.2 Molecular targeting strategies

Therapeutically, SERPINE2 represents an attractive but still early-stage target, because it may influence both tumor-intrinsic stress adaptation and extracellular microenvironmental remodeling. One mechanistically supported rationale comes from liver cancer, where SERPINE2 stabilizes EGFR by inhibiting c-Cbl-mediated ubiquitination and receptor degradation, thereby sustaining downstream oncogenic signaling. In this context, SERPINE2-directed intervention may potentially complement EGFR-targeted therapy by limiting compensatory receptor stabilization and restoring receptor turnover, rather than simply duplicating conventional receptor blockade. In addition, emerging evidence from radioresistant lung cancer models suggests that SERPINE2 may participate in ATM-associated DNA damage response signaling and homologous recombination repair, raising the possibility that SERPINE2 inhibition could sensitize selected tumors to radiotherapy or other DNA-damaging treatments. However, this DDR-related therapeutic implication should currently be viewed as tumor-context-dependent and not yet broadly validated across cancer types (11). A second therapeutic dimension relates to the extracellular and stromal functions of SERPINE2. Multi-omics and stromal-focused studies in gastric and colorectal cancer support a model in which SERPINE2 contributes to CAF-associated remodeling, macrophage polarization, and immunosuppressive niche formation (14). These observations suggest that extracellular SERPINE2 blockade may be particularly relevant in stromal-rich or immune-excluded tumors, where the goal would be not only to impair metastatic support functions but also to improve microenvironmental accessibility for other therapies, including immunotherapy. At present, however, these combination concepts remain biologically plausible rather than clinically established.

From a drug-development perspective, SERPINE2 appears more tractable as a secreted and extracellularly accessible target than as a classical intracellular oncogenic enzyme. This suggests that the most realistic initial modalities may include neutralizing antibodies, ligand-trapping biologics, or agents that disrupt key extracellular interactions. By contrast, if future studies demonstrate that tumor-promoting SERPINE2 functions also depend on intracellular expression programs or broader signaling-associated effects, alternative strategies such as RNA-based silencing or antisense approaches may be required. Importantly, no clinically established SERPINE2-specific inhibitor is currently available, and the translational feasibility of direct SERPINE2 blockade therefore remains to be demonstrated (31). In addition, because SERPINE2 participates in extracellular protease balance and tissue remodeling, potential on-target effects on normal stromal homeostasis, wound repair, or vascular remodeling should also be considered during future therapeutic development. Overall, the current literature supports SERPINE2 as a druggable conceptual target, but not yet as a clinically mature one. Future progress will depend on matching therapeutic modality to biological context, distinguishing extracellular versus tumor-intrinsic SERPINE2 functions, and validating whether SERPINE2-targeted intervention can enhance the efficacy of EGFR-directed therapy, radiotherapy, or immunotherapy in appropriately selected tumor settings.

Despite its therapeutic appeal, SERPINE2 targeting remains at an early and largely conceptual stage, and several translational issues should be considered more explicitly. First, there are currently no clinically established SERPINE2-specific inhibitors, and the most realistic drug modality remains uncertain. Given that SERPINE2 is a secreted protein with important extracellular functions, neutralizing antibodies, ligand-trapping biologics, or extracellular interaction-blocking agents may represent the most feasible initial strategies. In contrast, if tumor-promoting effects of SERPINE2 also depend on intracellular or signaling-associated functions, these may require distinct approaches such as RNA-based silencing, antisense oligonucleotides, or targeted protein suppression strategies. Second, on-target toxicity will need careful evaluation. Because SERPINE2 is a secreted serine protease inhibitor involved in extracellular proteolytic balance and tissue homeostasis, systemic inhibition could potentially affect normal stromal remodeling, vascular integrity, wound repair, or inflammatory resolution. This suggests that therapeutic windows, tumor-selective delivery, and context-specific patient selection will be critical for successful translation. Third, it remains unclear whether all SERPINE2-driven tumor phenotypes would be equally targetable with a single intervention. Tumors in which SERPINE2 acts predominantly through extracellular matrix remodeling or macrophage reprogramming may be more amenable to extracellular blockade, whereas tumor-intrinsic effects linked to receptor stabilization or stress adaptation may require combination approaches or deeper mechanistic stratification. Accordingly, future translational development should prioritize not only inhibitor discovery, but also modality matching. A practical therapeutic framework may require distinguishing whether SERPINE2 functions primarily as an extracellular stromal mediator, a tumor-cell-associated signaling facilitator, or both in a context-dependent manner. Such considerations are essential before SERPINE2-targeted strategies can move from conceptual rationale to realistic clinical development.

### 5.3 Immunotherapy combination opportunities

The immunological implications of SERPINE2 targeting are particularly significant. By promoting M2 macrophage polarization and contributing to stromal activation, SERPINE2 fosters an immune-suppressive microenvironment characterized by reduced cytotoxic T-cell infiltration and enhanced immune exclusion. Inhibiting SERPINE2 may therefore help reverse macrophage polarization toward a more pro-inflammatory phenotype and restore antitumor immune responses. Furthermore, SERPINE2-driven extracellular matrix remodeling may create structural barriers that limit T-cell penetration into tumor nests. Targeting SERPINE2 has the potential to reduce matrix density and improve immune cell accessibility, thereby enhancing the efficacy of immune checkpoint inhibitors. In tumors exhibiting high SERPINE2 expression and immune “cold” phenotypes, combination therapy with SERPINE2 inhibition and anti-PD-1/PD-L1 blockade may represent a promising strategy to overcome primary or acquired resistance. Taken together, these therapeutic perspectives position SERPINE2 as a multidimensional target in the multi-omics era. By simultaneously influencing oncogenic signaling persistence, genome integrity maintenance, stromal architecture, and immune exclusion, SERPINE2-directed interventions may offer a novel avenue for improving treatment outcomes in advanced cancers.

## 6 Conclusions and future perspectives

Collectively, current evidence supports a model in which SERPINE2 functions as an integrative regulator bridging tumor-intrinsic signaling and microenvironmental reprogramming. By stabilizing oncogenic receptors such as EGFR, enhancing DNA damage response pathways through ATM activation, and orchestrating stromal and immune remodeling, SERPINE2 coordinates multiple hallmarks required for metastatic persistence. Its dual role within cancer cells and the surrounding microenvironment underscores its unique position as a molecular hub in advanced disease. Importantly, multi-omics approaches have been instrumental in revealing the context-dependent yet convergent functions of SERPINE2 across cancer types. Transcriptomic, single-cell, methylomic, chromatin, and proteomic analyses consistently rediscover SERPINE2 in metastatic, stromal-rich, and therapy-resistant tumors. Although the cellular source and dominant downstream pathways may vary between tumor types, the overarching theme remains consistent: SERPINE2 supports metastatic adaptation by synchronizing signaling persistence, genome maintenance, and immune modulation. This convergence across independent omics layers strengthens the biological plausibility of SERPINE2 as a key driver of post-metastatic ecosystem remodeling.

At the same time, several important limitations in the current field should be acknowledged. Much of the mechanistic evidence supporting SERPINE2 function is still derived from *in vitro* systems or animal models, whereas large-scale clinical validation in independent patient cohorts remains limited. As a result, the translational relevance of SERPINE2 as a biomarker or therapeutic target has not yet been fully established. In addition, although SERPINE2 is recurrently identified across multiple omics platforms and cancer types, its dominant biological roles may not be identical in all tumor contexts. In some cancers, SERPINE2 appears to function predominantly through tumor-intrinsic programs such as receptor stabilization and therapy adaptation, whereas in others its strongest associations are observed in stromal remodeling, macrophage polarization, or immune exclusion. This heterogeneity suggests that SERPINE2 should not be interpreted as a uniformly acting effector across all advanced cancers, but rather as a context-dependent regulator whose functions may vary according to tumor type, cellular source, and metastatic niche. Nevertheless, these limitations do not diminish the significance of SERPINE2; rather, they define the key priorities for the next phase of investigation.

From a therapeutic standpoint, targeting SERPINE2 may represent a conceptual shift in the management of advanced cancers. Rather than focusing exclusively on oncogenic mutations or isolated immune checkpoints, SERPINE2-directed strategies aim to disrupt a coordinated survival network that integrates receptor stabilization, DNA repair enhancement, stromal activation, and immune exclusion. Such an approach aligns with emerging paradigms in precision oncology, where multi-layered molecular vulnerabilities are targeted simultaneously to overcome adaptive resistance. Nevertheless, several critical questions remain. First, spatial multi-omics validation is urgently needed to delineate the precise cellular sources and microanatomical distribution of SERPINE2 within metastatic lesions. Integrating spatial transcriptomics, multiplex proteomics, and imaging-based platforms will clarify how SERPINE2 expression correlates with immune exclusion zones, fibroblast-rich compartments, and vascular remodeling *in situ*. Second, the development of specific SERPINE2 inhibitors—whether small molecules, neutralizing antibodies, or biologics capable of disrupting key protein–protein interactions—remains a priority. Preclinical models evaluating pharmacologic blockade are essential to determine therapeutic feasibility and safety. Third, prospective clinical stratification studies are required to establish whether SERPINE2 expression or circulating levels can predict treatment response, particularly in the context of radiotherapy, targeted therapy, and immune checkpoint blockade.

In summary, SERPINE2 emerges from multi-omics investigations as a multifunctional regulator of metastatic progression and immune evasion. Continued integration of spatial and longitudinal omics data, combined with translational drug development efforts, will determine whether SERPINE2 targeting can move from conceptual promise to clinical reality in advanced cancer management.
